# Staying isolated indoors means that nobody sees me”: ontological (in)security and living with significant appearance concerns before, during, and ‘since’ COVID-19

**DOI:** 10.1080/17482631.2024.2374779

**Published:** 2024-07-03

**Authors:** Christian Edwards, Berenice Mahoney, Emma V. Richardson, Beck Lowe

**Affiliations:** a School of Sport and Exercise Sciences, University of Worcester, Worcester, UK; b School of Psychology, University of Worcester, Worcester, UK

**Keywords:** COVID-19 legacy, ontological (in)security, significant appearance concerns, qualitative, healthcare support

## Abstract

**Purpose:**

Though a worldwide period of uncertainty (COVID-19) has ‘ended’, there exists a legacy of maladaptive experiences among people with significant appearance concerns (SAC) that requires care and attention.

**Methods:**

Using Giddens’ concept of ontological security, we explored how people experienced their SAC before, during and “since” COVID-19. Qualitative surveys allowed us to capture diverse perspectives from individuals transnationally, analysed with deductive reflexive thematic analysis using ontological security as our theoretical foundation.

**Results:**

Themes named “More Mirror(ed) Time” and “Locked Out, Shut Down, and Shut Out” gave a contextual grounding for the embodied experiences of this group through times of social restrictions, and the theme “Redefining Relevance” explored the continued legacy of COVID-19 – and continued global uncertainties such as economic hardship and warfare – that impact the wellbeing of people with SAC.

**Conclusions:**

People with SAC are still ‘locked out’ from essential healthcare support as those providing healthcare are overworked, under-resourced and rely on efficient interactive methods such as tele-health that may be triggers for people with SAC. Care providers may consider expanding appearance concerns verbiage, look to involve trusted others in the care-seeking process, and utilize modalities beyond digital health to support people with SAC.

## Introduction

The start of the 2020s saw a drastic societal shift due to the global pandemic of coronavirus disease 2019 (COVID-19). Continuing legacies of healthcare disruptions, economic and ideological uncertainty, and austerity-driven policies have been destabilizing on a global scale, their life-altering effects felt long after the virus itself was reportedly on the decline. The intermittent implementations of lockdowns were particularly disruptive, connected to increases in anxiety, depression, trauma, emotional exhaustion, and existential dread (Quigley et al., 2023). For those vulnerable to body image concerns or eating disorders (ED), these periods of change accentuated a common thread of uncertainty that continued to impact how they managed and received care for their condition(s), even after restrictions were lifted (Bannister, 2021; Miller et al., 2022; Shaw et al., 2021; Syed et al., 2022; Trott et al., 2021). The prevailing theme of uncertainty amid disruption among those with image and eating concerns warranted further exploration, inspiring a theory-based approach to capture the life-altering effects of those who are unified by insecurities pertaining to identity, the body, and the self.

Our research centred on individuals with symptoms akin to Body Dysmorphic Disorder (BDD) during disruptive events (i.e., the COVID-19 pandemic). In the Diagnostic and Statistical Manual of Mental Disorders (DSM-5), BDD falls under the umbrella of obsessive-compulsive and related disorders (American Psychiatric Association [APA], 2013). Individuals with BDD are preoccupied with perceived defects or flaws in their physical appearance and these concerns cause significant distress or impairment in functioning (APA, 2013). Given, however, that BDD may share some of its symptomologies with ED and exercise addiction (Milligan & Middleman, 2022; Tremblay et al., 2019), clinicians, and people living with these preoccupations, may not recognize or differentiate its/their symptoms from other related disorders (Murray & Baghurst, 2013; Phillips, 2005). A person with BDD may live with another eating or appearance-related disorder, and though symptoms may co-occur, both disorders require separate and specific diagnosis and treatment (Phillips, 2005). Individuals also construct their own internal narrative such that their ontological reality of appearance concerns may not be in-line with a specific diagnosis (Pellegrini et al., 2021). Further, some individuals may experience significant distress and impairment surrounding aspects of their physical appearance, but do not yet have a formal clinical diagnosis or are “not sick enough” for a clinical diagnosis (Eiring et al., 2021; Murray & Baghurst, 2013; Nutley et al., 2021). To that end, in this study, we are inclusive of individuals outside the scope of a BDD diagnosis who nevertheless self-identify with its symptoms. Accordingly, in the current work, we use the term “significant appearance concerns” (SAC) to delineate inclusively the shared symptomatology of our population of interest; namely, individuals with a self-reported clinical diagnosis of BDD, sub-clinical BDD, and experiences individuals interpret as relating to BDD. Although the term SAC may not conform with the BDD diagnostic criteria (APA, 2013), previous research has used similar non-technical terminology because it may be more comprehensible to participants than medicalized terms (Fatt et al., 2021) and, for the current work, may reflect the ontological realities of those who self-identify with appearance concerns.

Prior scholarship indicates that social restrictions enforced during the COVID-19 pandemic may have exacerbated symptoms and maladaptive behaviours in those susceptible to SAC (Syed et al., 2022; Trott et al., 2021). Often this research has recruited disorder-specific community samples (e.g., individuals with ED) and these articles may not capture the full range of preoccupations and associated symptoms of the current sample (Linardon et al., 2022; McLean et al., 2022). Nevertheless, existing research suggests that individuals with SAC may have excessively turned to their bodies, corporeal practices, and more controlled eating/exercise to negotiate and gain security when faced with the uncertainty and adversity of the pandemic (Linardon et al., 2022; McLean et al., 2022). Research on sub-clinical samples also yielded homogenous results that made plausible a connection between enforced social restrictions and the worsening of SAC symptomology (e.g., Monthuy-Blanc et al., 2023). Building on this connection, we sought to fill the gap in qualitative research designated for (i) more diverse perspectives and (ii) theory-based approaches to better understand the nuanced manifestations of SAC since the arrival, and continuing legacy, of COVID-19. The present study harnessed the social restrictions era to meaningfully organize the many complex manifestations of SAC through employing the concept of *ontological security*, with a view to directing long-term, effective care from healthcare services.

### Ontological (In)security

As BDD sits with obsessive-compulsive disorders in the DSM-5, it is possible that these individuals may share a common need for self-control and perfectionism during periods of uncertainty (Pinciotti et al., 2021; Pinto et al., 2017). Broadly, this need for (self)control lends itself to the concept of ontological (in)security. Ontological security was coined by Ronald Laing who, influenced by existential psychology, defined it as experiencing oneself as “real, alive, whole, and, in a temporal sense, a continuous person” (Laing, 1990, cited in Gustafsson & Krickel-Choi, 2020, p. 881). Laing’s notion of continuity aligned with our interest in sustained experiences of the self before, during and “since” COVID-19 enforced social restrictions (e.g., lockdowns). For a person with SAC, though, it may be that their physical body serves as a controllable aspect of their sense of self and, thus, their nexus to ontological security when life around them is uncertain. This notion may speak more to Anthony Giddens’ conceptualization of ontological (in)security, whereby an individual’s sense of identity, agency, social stability, and reality relies on the continuity of routines (Giddens, 1991). Without them, “chaos lurks” (Giddens, 1991, p. 36) augmenting a sense of ontological insecurity. Giddens asserted that ontological security is thwarted when the body becomes invisible or is disconnected from the self (Giddens, 1991). Importantly, the appearance-related self-reflexivity that those self-identifying as living with SAC may engage in to maintain their ontological security could make disruptions to appearance work more salient (Giddens, 1991). This serves as a crucial component of the present study as Giddens’ concept of ontological security has the potential to help understand the conflict between an individual’s evaluation of their own vulnerability, and the belief that such vulnerability is relevant and worthy of support.

Some research has already identified the ways ontological security was threatened by the removal and subsequent loss of control (personal autonomy and freedoms, community engagement, witnessing of inconsistent policymaking and enforcement) due to compulsory COVID-19 regulations used to “tackle” the pandemic (Mamzer, 2020). COVID-19-related lockdowns, for example, are reported to have elevated anxiety and depression levels (Dettmann et al., 2022), particularly in those already vulnerable to these mental health outcomes (Andersen et al., 2021; Husky et al., 2021), and consequences worsened for individuals with less social support and economic security (Dickerson et al., 2022). Moreover, the (re)introduction of lockdowns exacerbated anxiety and depression levels (Quigley et al., 2023), emphasizing the need for a certain, controllable, and sustainable routines for the continuation of ontological security. More specifically to SAC, with the arrival of multiple external instabilities (e.g., the COVID-19 pandemic), it may be the case that the body (and body work) acts as a control strategy to (re)gain a sense of continuity (Shilling, 2013). For example, during COVID-19, some individuals with ED relied on food as “an auxiliary coping mechanism” after lockdown measures were instigated (Branley-Bell & Talbot, 2020). Then again, for other individuals, the forced change in routine because of COVID-19 lockdowns may have offered them temporary relief allowing for respite from potentially triggering situations (Miller et al., 2022). It is, therefore, important to consider that social restrictions may have “benefited” some people with SAC by prompting them to relinquish control, retreat from social roles and re-evaluate the factors that were once integral to their ontological security. However, heterogeneity regarding the impact of social restrictions on individuals living with diagnosed or self-identified SAC is less considered in the research.

It is also possible that perceived support, relative to the actual support available, plays a part in the conflict between SAC and ontological security. Individuals with SAC reported facing barriers to healthcare services and social support due to their concern for others, denial of symptoms, perceived stigma, shame, fear of losing control, and the belief that their symptoms did not warrant support (Ali et al., 2020; McCausland et al., 2021). At the same time, healthcare services have experienced, and continue to experience, the impact of the pandemic (Bannister, 2021; Shaw et al., 2021). Taken together, it is troubling that those who perceive their SAC to be irrelevant and unworthy of support could have their beliefs substantiated by the lack of resources available to them.

### Study aims

The present study used Giddens’ concept of ontological (in)security to explore how people experienced their SAC before, during and “since” COVID-19. In the current work, we aimed to accommodate the perspectives of those who reported significant concerns about any aspect of their appearance, body shape and size, or who had been diagnosed with BDD.

## Method

The study methodology was informed by interpretivism. Variants of interpretivism exist (Alharahsheh & Pius, 2020), but its fundamental tenets continue to provide qualitative researchers with a “broad epistemological view” (Howe, 1998, p. 13). Interpretivism fundamentals treat subjectivities and the social world as drivers of how individuals experience realities that are deemed contextual, relative, and thus uncertain (Howe, 1998). Individuals construct their realities through their interpretations, active meaning and sense making of the self and identity in contexts and events that can be changeable (Howe, 1998). This “broad epistemological view” instantiates the ontological security concepts used in this study. Specifically, the individual’s sense of identity, agency, social stability, and reality; *ergo* their ontological security is tied to the continuity and certitudes of routines in their life (Giddens, 1991). Disruption of these routines prevents the continuity of one’s self-identity and may erode and destroy ontological security, resulting in anxiety and the potential for personal chaos and crisis (Giddens, 1991).

Given the context of COVID-19 lockdowns in place when the data were collected, an online qualitative survey method was used to capture individual’s experiences of their SAC-related behaviours and beliefs, symptom severity and support needs before, during, and “since” the arrival of COVID-19. Internet-based methods, including qualitative online surveys, can also provide a safe, virtual and ethical space for engaging vulnerable groups in research (Neville et al., 2016). The online qualitative survey method can be advantageous over face-to-face, one-to-one interviews when researching the experiences of such groups, defined here as those at “ … increased risk or susceptibility to adverse health outcomes” (Flaskerud & Winslow, 1998, p. 69). For example, being asynchronous, with minimal researcher involvement, online qualitative surveys offer participants a level of anonymity, control, and autonomy over face-to-face, one-to-one interviews. Increasingly, this internet-based method is used to capture rich data of diverse perspectives and experiences from vulnerable groups on sensitive topics related to their physical and mental health (Braun et al., 2021; Neville et al., 2016). Individuals who believe they have SAC, with or without a clinical diagnosis of an appearance-related disorder, fit this definition of “vulnerable”, and are relatively unknown, likely to be diverse, and thus hard to access. Importantly, greater self-consciousness about appearance and perceived stigma regarding their SAC could help these individuals feel more comfortable participating in an online survey than face-to-face interviews (Braun et al., 2021). The online survey method also facilitates recruitment from socially and geographically diverse populations (Braun et al., 2021; Neville et al., 2016). Braun and colleagues’ (2021) best practice guidance for online qualitative surveys informed development of the study survey which comprised of a series of open-ended questions with free-text response boxes created using surveyhero.com (TM). The questions asked participants to describe their SAC and help-seeking for these concerns before, during, and “since” the arrival of COVID-19. Where appropriate, brief stimulus materials (Association of Qualitative Research, n.d.) were used to facilitate depth and breadth, and avoid perfunctory, qualitative responses (Braun et al., 2021; e.g., DSM-5 Body Dysmorphic Disorder specifiers).

### Positionality

All contributors to this research had direct and/or indirect lived experience of eating disorders and other body-related preoccupations (e.g., eating disorders, and muscle-orientated preoccupations). We brought these experiences to interpretations of results akin to the requirements of reflexive thematic analysis (Braun & Clarke, 2019), acting as sounding boards and critical - but supportive - friends. Further, each researcher brought their own distinct areas of specialism including body-image concerns, qualitative methods, and health care research to the research. Throughout the process we discussed our positionality as it related to our interpretation of the data, ensuring we identified interpretations that were inadvertently influenced by our own contexts and biases.

### Recruitment and data collection

Sixty-nine participants were recruited to the study and their data were collected from July 2020 to March 2021. A flyer was posted on the online research portals of two leading UK based charities supporting individuals with SAC, and the following social media platforms (Facebook groups, Instagram; researcher Twitter accounts of Christian Edwards and Bere Mahoney). The flyer called for “men and women aged 18 years or older who have significant concerns about any aspect of their appearance, body shape and size, or who have been diagnosed with Body Dysmorphic Disorder (BDD) to complete an online survey”. Potential participants accessed the online qualitative survey through either the hyperlink or QR code on the flyer. In the online survey portal, potential participants were first presented with an information sheet, followed by a consent form. The information sheet assured them that participation was voluntary, that they could skip questions they did not wish to respond to, and that they could withdraw from the study at any point whilst completing the online survey by closing their web browser and that their responses would not be stored. To maintain anonymity, they were also advised that once they had submitted their responses they could not withdraw their data from the study. Individuals indicated their consent to participate using checkbox responses that confirmed they had read and understood the information sheet, that their participation was voluntary and that they were 18 years of age or older. Only individuals who endorsed all these statements were deemed to have given consent to participate and were then automatically taken to the online survey. The scales and questions we used for the survey are available in the Appendix. The sample mean age was 31 years (SD = 11) and the majority described themselves as female (71%), white (82%), and educated to degree level or higher (65%). Most described their nationality as British or “UK” (58%) and reported living within the UK, but the sample included at least one national and resident from Europe, North America, Australasia, South Asia, and the Far East.

In the UK, the first mandated lockdown was announced by the government in July 2020 and the second in October 2020. In March 2021, the “stay at home order” ended and there was a reopening of schools, outdoor sports facilities, and outdoor social gatherings were permitted (see https://www.instituteforgovernment.org.uk/data-visualisation/timeline-coronavirus-lockdowns). Given our focus, we collected data during these lockdown periods (i.e., the survey opened in July 2020) until the “stay at home order” was lifted in March 2021 (i.e., the survey closed at the end of March 2021). We decided to “stop” data collection based on pragmatism regarding time and resources of the researchers, and the willingness of participants to participate (Braun & Clarke, 2021). That is, we kept the survey open for as long as possible to allow participants ample time to complete. We decided to end data collection when there were no additional responses after 3 weeks, despite saturating the portals of the UK-based charities and researcher social media platforms for a period of 10 months. We judged at that point that those willing and comfortable to share their stories had done so.

### Data analysis

Reflexive thematic analysis was used to analyse survey responses (Braun & Clarke, 2021). Our interpretivist, theory-led approach warranted a latent analysis, whereby implicit meanings were derived from the data and juxtaposed with the positionality of the researcher. As such, reflexive thematic analysis and hybrid, line-by-line coding were used to analyse survey responses (Braun & Clarke, 2021). We (Christian, Berenice and Emma) initially immersed ourselves independently in the data by familiarizing ourselves with the free-text responses, identifying preliminary codes inductively to ensure positionalities and subjectivities were accounted for. With preliminary codes confirmed, each researcher subsequently used the concept of ontological (in)security to explore the data deductively. We discussed numerous different theories and concepts from our respective fields as potential lenses but chose ontological (in)security as we judged this lens added essential meaning and depth to the what, why, how and so what of SAC during and after lockdown. Deductive coding was utilized to critically explore emergent themes through our specific theoretical lens, verifying the applicability of the lens in the context of the phenomenon in question and providing replicable criteria for future researchers. To ensure rigour, a fourth researcher (Beck), with direct experience of SAC during lockdowns, was invited to interpret the research after initial themes had been identified, confirming that the research approach was impactful by way of attributing a time-specific phenomenon to a historic, sustainable concept. To further enhance rigour, resonance and potential transferability of findings to applied settings, we presented our findings to a diverse group of stakeholders including health care practitioners, applied researchers in this area, and qualitative research specialists. Stakeholder reflections highlighted the resonance of the themes with regards to their professional experiences, but also illuminated aspects of findings we had not considered. As a result of the reflexive discussion, we amplified findings on the salience of digital means of support, and the heterogeneity of SAC experiences “since” COVID-19.

### Ethical approval

Ethical approval for the study was given by the relevant institutional Ethics Panel (21/7/2020, *REP CODE: CBPS19200034-R*). We adhered to ethical handling of the data and ensured the confidentiality of participants by removing identifiable characteristics (e.g., name).

## Results: ontological (in)security

Our findings were connected by a common thread of ontological (in)security during times before, during and “since” COVID-19; effectively, the everyday experiences of participants and how they were able to either maintain a sense of security, renegotiate their sense of security, or felt insecure during this time. We crafted three key themes developing this idea further by identifying how individuals perhaps “did” ontological security or what ontological (in)security might look like for people with SAC. The themes “More Mirror(ed) Time” and “Locked Out, Shut Down, and Shut Out” provide a contextual grounding regarding the experiences of persons with SAC before, during, and “since” the arrival of COVID-19. We explore the impact of these experiences in our last theme “Redefining Relevance”. Thereafter, we discuss why such findings are relevant in today’s “post-COVID” world, and the ongoing psychological and social burdens that affect participants. We provide recommendations and implications of practice as a concluding element of this work.

### More mirror(ed) time

During the time data were collected, the world moved online; the “real” world was lived through a virtual medium with meetings, classes, appointments and social interactions with family and friends happening on screen rather than in person. In the UK, for example, individuals were required to stay in their homes for significantly long periods and were often required to appear on camera. As a participant explained; “I have to use video call more for work and the camera always looks different to the mirror. The camera seems to present a different version of me … [a] distorted version, and leaves me wondering—which is the truth of how I really look, the camera me or the mirror me (or neither)?” (Female, 33, British). For many participants in our study, this extended screen time eroded their ontological security and negatively impacted their sense of self. In turn, to protect their ontological security, they sought alternative ways to enhance control of their appearance. For example, some individuals felt they had to manage their appearance to the extent of someone who appears on screen professionally. A new urge for some participants was to “prepare” settings to appear as their most unflawed self; “*video calling—I would never have done that before, given the choice. I don’t like how I look in the camera, I have to ‘prep’ in advance with lighting, camera angles, having a mirror nearby, make up on hand, hair done etc.”* (Female, 33, British)

Even those who were not required to use webcams were forced into situations where anxieties around appearance were emphasized. Some participants, for example, described their normal security method was to avoid their reflection; *I don’t spend time looking in the mirror just the opposite I avoid reflections wherever they occur.”* (Male, 68, British). Being stuck at home, however, meant they were exposed to more mirrors and had *“more time at home to be able to mirror check.”* (Female, 55, British). As this participant articulates; “ … *I walk past mirrors and catch my reflection more when working from home. Pre COVID-19 I would be out and about for work and would hardly see my reflection during the working day*.” (Male, 29, British). For these participants, the result of being confined to home often resulted in them “doing” security by becoming more consumed with checking their mirrored appearance. A participant, for example, described how she “did” security by compulsively inspecting her face; “*I usually avoid my reflection. Being stuck at home, looking in (the) mirror loads more and studying my face, black heads, hairy face, awful eyebrows, marks, horrible teeth, fat; depression about these things.”* (Female, 35, British).

For many participants, the usual coping strategies for managing their ontological security were taken away which resulted in many individuals experiencing anxiety and a loss of control. For example, this participant discussed being able to manage mirrors before COVID-19-related restrictions as her outlet was the gym. However, when her coping strategy of exercise was taken away, she turned to build her ontological security through compulsively (perhaps addictively) checking her appearance in her home mirror. She felt such an experience seriously undermined her mental health:

Before COVID-19 I worked out 2–3 times a week, now I haven’t been in my gym since they reopened. Working out is the absolute best medicine for me, but now, when I haven’t been up there for over 3 months! I’m freaking out and … terrified of starting again due to my anxiety towards people and the mirrors. Before the COVID-19 I still hated what I saw in the mirrors but I managed, now because I’ve been “locked in” for so long my “bag” has worsen[ed] to a degree [that] I never experienced before. I’m stuck to the one mirror I can look at myself in, but addicted to checking my features and skin more than 100 times a day. I can(n’t) get out of the black whole/circle … I’m very suicidal. (Female 35, Denmark)

For many participants, mirrors were a key trigger to their appearance concerns—something they did not want to look at yet felt compelled to—that heightened anxiety and poor mental health. For individuals required to appear on screen for work, education or medical reasons, such insecurity was exacerbated because “lock down” forced a significant change in social interactions.

### Locked out, shut down, and shut out

Expanding on the notion of being “locked down”, this theme encompasses the complex and diverse experiences of being “locked down” as a society, ongoing support and therapy being “shut down” and replacement therapy being unsuitable or non-existent such that participants felt “shut out”. The complexity of lockdown is exemplified in the following quotes as some individuals, rather than experiencing a heightened anxiety from more mirror or screen appearance time, found this period to be a positive experience because mandated restrictions may have served to protect their ontological security:

The COVID-19 lockdown experience is compatible with my social anxiety and appearance-anxiety since staying isolated indoors means that nobody (except immediate family) sees me. Due to not having to go outside to attend to outdoor social obligations (because of lockdown closing all non-essential services/programs and my full-time college degree academia becoming a virtual program), I have been able to relax my commitment to conforming to beauty standards by no longer wearing make-up or feeling highly stressed about getting dressed to go outside. I stay indoors, bare-faced, no make-up, in casual informal indoor at-home attire and self-seclude in my own room unless I absolutely have to leave it. (Female, 25, Canadian)

Others described how enforced mask-wearing served to protect their ontological security.

Going out in public is easier for me now [during the current restrictions] because I have to wear a mask. Covering my face gives me comfort. Before COVID, walking down the street with my head held high was impossible because I felt that people would judge me and find me too ugly to look at. (Female, 50, England)

However, while some participants experienced almost a respite by wearing face coverings or excessively managing their appearance, many were negatively impacted as the therapeutic ways they controlled their appearance concerns were “shut down”. This included: (i) being unable to access medical support for skin conditions; *My acne took a very sudden inflammation and flare up which require me to seek desperate help from my GP for further dermatologist review after a dermatologist appointment was cancelled and access to general review was removed* (Female, 31, British), (ii) termination of therapy or counselling; “*My therapy has been pretty much completely cut off. I get one phone call for 10 minutes every 3 months. Can’t get a GP appointment either as my local doctors is already fully booked weeks and weeks in advance.”* (Female, 27, British); *… [I] was discharged from CMHT (community mental health team) for some reason during lockdown. [I’ve] been rereferred, [and am] waiting to hear from them”. (Female, 27, England)*, and (iii) restrictions on other therapeutic and supportive contexts; *“COVID came along at a time when I was going to the gym and going to see a personal trainer for help. My gym has been closed since March and has not re-opened yet.”* (Male, 28, British); ‘ *… Not being able to do my usual gym routine has made my thoughts stronger about not looking the way I want to*.’ (Female, 27, British). The result for many individuals was an overreliance on other corporeal-oriented ontological security management strategies and a sense of deterioration in their mental health; *During lockdown, spent more time exercising than normal. [My] mental health deteriorated and [I] felt trapped. [Exercise] helped.”* (Female 35, UK); *[I] have become a bit more obsessive about calories due to not being able to workout.”* (Female, 27, British); *“During COVID, I developed an eating disorder. This has made me feel more indifferent to my body … . [and] means I feel even more … that I am not slim enough.”* (Female, 18, British).

While some participants were referred for counselling and support, the necessity to use digital health care (e.g., telehealth, e-health, m-health, online support), left many participants feeling “shut out”; *I cannot visit either of my therapists, their treatments are very hands on so virtual meetings are not possible*.” (Female, 27, British). Arguably, this digital method of counselling may have also heightened anxieties of appearance on screen (highlighted in ‘More Mirror(ed) Time); “*I’ve struggled to find support forums that don’t involve being on Zoom or something similar.”* (Female, 55, England).

These first two themes set the scene for what people with appearance concerns experienced during COVID-19 restrictions. What is arguably, more significant to understand is how such experiences impacted participants’ relationships with themselves and other people.

### Redefining relevance

Within the underpinning grounding of ontological (in)security, we identified four ways in which participants experienced changes in what and who were relevant during the time of the pandemic. These were (i) “I am irrelevant”, (ii) “My worries are irrelevant”; (iii) “Who is relevant?”; and (iv) “What is relevant in my life?”

#### ‘I am irrelevant’

First, some participants perceived themselves or their problems as not relevant compared to COVID-19. For example, these participants perceived their worries were now insignificant; “*I feel like I can’t go to the doctor, and I can’t speak about my worries as much because I know that there are bigger and worse problems going on around me. My worries seem massively insignificant compared to this [pandemic].”* (Female, 22, British); *With the risk of covid-19, I don’t feel professionals have the time [for me].”* (Female, 50, British); *“I feel like it [my concerns about myself] would be disregarded [at the moment] due to the amount of backlog currently in the system.”* (Female, 22, British).

#### ‘My worries are irrelevant’

Second, from a more positive perspective, some participants redefined the relevance of whether their physical appearance was something to worry about during this time: *“I honestly feel less worried about my appearance. Everyone’s struggling, and I encounter a lot fewer people in a day than I used to. No need to be self conscious in my own home with nobody around but my partner.”* (Female, 23, USA); “[During this time] I have become more confident in my appearance. I have challenged my beliefs that ‘I am too short’ and that ‘I am fat’. I still want to be thin (even though my BMI is 19) and I want to be skinnier. I keep wanting to always improve and lower my weight, but at least I am aware of this and can keep it in check.” (Female, 23, Australian).

#### ‘Who is relevant?’

Third, some participants stated that COVID-19 restrictions gave them time to reflect on their relationships with others, and reconsider who brought a positive aspect to their life:

“Before COVID-19 I hung around people who were not happy for my success and I had a lower self-esteem. COVID gave me the time and space to think about who I need in my life. I am more self-aware now and can recognise when someone is not ‘rooting’ for me. I prefer to be associated with people who celebrate my accomplishments rather than bring them down.” (Female, 23, Australian)

“I have spent more time with my self and the choices I make daily. Every day is a new day and I am grateful. I am able to be more selective on how I spend my time and who I spend it with.” (Female, 51, USA)

#### ‘What is relevant in my life?’

Fourth, some participants experienced a very mixed redefinition of what was relevant in their life.

I think Covid has made me more aware of what I can and can’t do e.g. visiting different places or people. Before lockdown I stuck to the same routines and habits, but during/now I try and change things up, start ticking off things on my list of things to do or visit because it’s made me realise that you might not have the time or physical capacity to do it in the future. (Female, 23, British)

For some, this redefinition encompassed a messy consideration of their own struggles while also feeling more fortunate than others:

I wasn’t feeling at my best before COVID. Lockdown increased me feeling stuck. It caused a lot of stress at home as there was increased time spent with my partner who was also stressed. So I was going to work to hold therapy sessions for patients who were and still are feeling very vulnerable and at the same time coming home to a very stressed environment where I couldn’t switch off. I felt like I didn’t have much choice in the situation. I started feeling more incompetent at work. Started wishing I could quit all my work but then at the same time reminding myself I just needed a break. The pressure of hearing of people losing their work because of COVID made me realise how lucky I am but at the same time part of me wanting to just break away from it all. (Female, 33, British)

The experiences described thus far reflects the time-period of restrictions between 2020 and 2021, however, the legacies of these experiences are ongoing and feeds into necessities of care in contemporary times.

## Discussion: implications for ‘post-COVID-19’ contemporary care

As far as we are aware, this is the first study using the concept of ontological (in)security to interpret the legacies of COVID-19 for people living with SAC. Our results show what ontological (in)security looked like for participants and reflect the complex ways participants negotiated their SAC within a period of significant disruption. Uncertainty, however, is ever-present, and ever evolving. Though COVID-19, from the standpoint of implemented social restrictions, may be over the legacy of these restrictions remains for many people with SAC. The body and corporeal practices may continue as the one “thing” that people perceive they may have complete control over, particularly as globally we have moved from one era of uncertainty (COVID-19) to another (economic crises and times of war). An appreciation of how individuals experienced their respective SAC before, during and “since” COVID-19 provides a comparative picture of behaviour and coping strategies that can help this community and health care providers work together to create a support structure that accommodates the needs of both parties. Thus, from our findings, we propose the following implications and contributions regarding the ongoing care and support of people with SAC.

### Continuous uncertainty

Insights from the current paper may be valuable for researchers and practitioners working with people with SAC, broadening the theoretical and conceptual menu they can draw upon to interpret how individuals’ appearance-related preoccupations may be influenced by, and be a response to, their perceptions of global, regional, local, and personal uncertainty. Our novel theoretical lens of ontological insecurity, and in particular Giddens’ concept of continuity, may provide a more holistic perspective on SAC and the legacies of COVID-19. Research exploring the phenomena of SAC during the pandemic may have viewed COVID-19 lockdowns as a single event or provided a snapshot of that timepoint (e.g., Branley-Bell & Talbot, 2020). However, as we have shown with SAC in the era of “post” COVID-19 social restrictions, a theoretical appreciation of SAC may be needed to understand the meanings of individuals’ concerns and support needs. The legacy of COVID-19 remains and impacts people with SAC regarding their sense of worth and access to support, uncertainty and perceived lack of control within ongoing global crises. As this work indicates, such a theoretical approach can illuminate changes in embodied practice and perceptions of self. Exploring SAC through the concept of ontological security, for example, enabled us to critique whether SAC shaped maladaptive behaviours such as compulsive mirror checking, or if COVID-19 restrictions merely “gave space” for SAC symptoms to manifest. In turn, this lens may broaden current perspectives that may often attribute SAC to individual, personal characteristics and social factors (e.g., personality attributes, perfectionism; for review see Phillips, 2005). While ontological (in)security is a potential theoretical lens that may be used to inform practice(s), it is advisable for researchers and practitioners alike revisit the process and development of individual SAC symptoms because such theoretical reasoning may move beyond the boundaries of a static presentation of symptoms (Pellegrini et al., 2021). For practitioners, also exploring and understanding how a person’s SAC may be tied to their fundamental sense of security could place them in a better position to support their needs in a meaningful way. Below, we expand on how these findings might inform practitioners and researchers working with individuals with SAC describing the following issues: (a) Amplifying Worthiness for Support, (b) Potential Movement from (Pre)Contemplation to Preparation and Action, and (c), Provision of Support.

### Amplifying worthiness for support

The (self)perception that people with SAC did not (and may still not) consider themselves a priority or worthy of care and support is ongoing. Participants’ stories revealed *why* they were reluctant to seek support and such motivations may be a beginning for health care providers to challenge the notion of not being worthy that remains, or has increased, since COVID-19 social restrictions “ended”. Indeed, ontological (in)security threats, such as ongoing global issues related to the cost-of-living crisis, austerity measures and warfare (Blokker & Vieten, 2022), have the potential to amplify the notion among people with SACs that the psychosocial significance of their issues do not matter on a grander scale. Organizations supporting people with SAC must be proactive in resisting discourse that there is a hierarchy of worth regarding those who need (or are worthy of) help. In resisting such discourse, organizations may look to, for example, validate symptoms and support needs, and visibly signpost this community to appropriate support.

We acknowledge that our findings represent people who self-selected to take part in the research. The findings and subsequent implications are therefore reflective of the perspectives of individuals who may already be interested and invested in help-seeking behaviours—or, despite potential inner turmoil regarding lack of worth, are receiving some degree of support. Given, however, that participants in the current study self-identified with SAC via an anonymous survey, some individuals may be experiencing denial or distress regarding their SAC and lack insight into their concerns (Bjornsson et al., 2010; Şenay & Yücel, 2022). As such, they may have not yet considered seeking help (Ali et al., 2020; McCausland et al., 2021). Nevertheless, we outline some potential implications from this research that may encourage such individuals to contemplate seeking help.

### Potential movement from contemplation to help seeking

Supplementing the verbiage of BDD to include something more descriptive and non-technical such as “significant appearance concern” may resonate more with individuals’ ontological reality and encourage them to seek help. In doing so, this may mitigate the intimidation of medicalized or jargonistic terms that act as barriers to help-seeking. Further, a more descriptive verbiage may help reduce the shame and stigma of being “labelled” with a mental health disorder (Fatt et al., 2021), such that people may seek help earlier and develop coping strategies to manage concerns and/or compulsions before the severity of symptoms escalate.

A further resource that may be helpful for people with SAC in accessing the help they deserve is through trusted others (Phillips, 2005). Within the “Redefining Relevance” theme, individuals with SAC sought support from individuals they had a secure relationship with, and these individuals may be the necessary signposts to professional help. Persons with SAC may trust the advice and perceptions of these individuals and may validate that they are worthy of help if encouragement comes from an external source (Migliorini et al., 2023). Outreach programmes may also wish to consider discourse that targets the spouses, family, and friends of persons with SAC as these gatekeepers may encourage and direct people with SAC to the formal support they require.

### Provision of support

An ongoing healthcare legacy of COVID-19 is the increased use and reliance on online digital health care to contact and interact with individuals. Considering participant experiences of “more mirror(ed) time”, this modality (for some) may act as a deterrent rather than an inclusive mode of communication for supporting people with SAC. That some participants sought to perfect their appearance for video calls was substantiated by the emergence of new terms for image concerns, including BDD (Türk & Jafferany, 2022), such as *video conferencing dysmorphia* (Sarangi et al., 2022) and *Zoom dysmorphia* (Jabali et al., 2023). Digital formats of health care may encourage cameras remain on during consultations for professional, ethical and care reasons, but for people with SAC, the presence of a peripheral reflection during online interactions could be at points counterproductive and harmful. It may be that health care professionals consider the options for camera-off consultations, or progressively introduce the use of cameras as part of their care.

Further, the provision of *only* digital health care to address the growing backlog of referrals for support may leave individuals feeling continually “shut out” from resources they have been directed to for support. Indeed, there is ongoing debate regarding (i) the effectiveness of digital healthcare as an equitable equivalent to in-person modalities for delivering care and support, and (ii) whether digital healthcare for mental health concerns has appropriate cross-diagnostic, condition severity, and cultural value (Gentile et al., 2018; Linardon et al., 2022). For some individuals living with SAC in our study, the specific meaning of being “shut out” suggests a potential misfit between digital mental healthcare and the support needs of individuals living with SAC and related disorders (Linardon et al., 2022; McCausland et al., 2021). In this sense, our findings counter broader research evidence optimistic about the trans-diagnostic acceptability and care experience of people with SAC regarding support via digital methods (e.g., Datta et al., 2020), and supports work that advocates for a more careful application of digital health among people with BDD (e.g., Türk & Jafferany, 2022).

We are not discounting digital health care as a mode of support. For example, digital health care may be the first step for reaching isolated individuals. Indeed, a key finding from this study was the respite some participants experienced as a result of social restrictions. For these participants, restrictions implemented such as lockdowns, limited contact, staggered re-openings of social spaces etc., were “compatible with” their SAC, and may have served in some way as a protective cocoon with which they continue to engage. The dangerous legacy of COVID-19 for these individuals is that the security, safety, and perceived break from their triggers may result in a self-imposed isolation or restriction of one’s engagement in the outside world, and digital health modalities are one of the few ways to reach these people and provide a gateway for further support that may progress from digital to in-person. Furthermore, the anonymity and control (if health care allows cameras to remain off) provided by digital health care modes may be a powerful motivator for people with SAC to engage in support. Indeed, Gough and Novikova (2020) reported that men were more likely to seek help for mental health difficulties if that modality of support permitted anonymity. Our critique of digital health modalities is therefore not that they do not have use, but that a reliance on these alone do not adequately serve all individuals with SAC (Enander et al., 2014; Türk & Jafferany, 2022). In other words, the heterogeneity of participant experiences of SAC before, during and “since” COVID-19, must be reflected in the provision of support in that this community may take different compensatory paths to support, and consequently support must be individualized according to the specific SAC a person brings with them.

The study findings should be interpreted considering these caveats. The study focused on the meaning of SAC for individuals with diverse self-identified appearance-related concerns. Using “self” not “others” identification of psychosocial concerns among community populations can mitigate sampling bias when recruiting to online COVID-related health research (De Man et al., 2021), and when conducting research that aims to include diverse, and often overlooked, individuals living with significant appearance-related concerns, with or without a clinical diagnosis of a related disorder (Cañate et al., 2021; McGrath et al., 2023). However, this strategy can be problematic, discouraging participation from some subgroups of individuals living with significant appearance concerns (e.g., those unsure of how to label their concerns, who question the significant appearance concerns and support worthiness, and who query the value of treatment, McCausland et al., 2021; Radunz & Wade, 2023; Schulte et al., 2020; those who fear stigma and feel shame about their concerns; Schulte et al., 2020; Stechler & Henton, 2022). Furthermore, our sample was international but comprised mainly of Western European and North American, white, well-educated females, common sampling biases in research on SAC (e.g., McGrath et al., 2023) and characteristics that can be associated with greater help-seeking for mental health problems but in ways that appear complex (Doll et al., 2021). Living with complex and severe significant appearance concerns and related conditions can also be a barrier to participating in research generally (Hughes-Morley et al., 2015). Thus, our findings may resonate more with individuals living with less complex and severe SACs, among the subgroups sampled, and capture their experiences of care and support for their SAC available within the country of residence.

## Conclusion

Using ontological (in)security as a lens to explore how people experienced SAC before, during and “since” COVID-19, we have shown that a continually uncertain world compounds this community’s perception of worth, a consequence of which can be coping strategies escalating and compromising health and quality of life. New considerations for care and support are warranted that reflect this group’s ontological realities. Providing multiple options for gateways to and pathways of care, such as expanding verbiage, engaging with trusted others, and modalities beyond digital health could be a start for supporting this group in a “post-COVID-19 world”.

## Data Availability

The data that support the findings of this study are available on request from the first author, (Christian Edwards). The data are not publicly available due to ethical protocols linked to confidentiality and anonymity of the participants.

## Author note

Edwards and Mahoney contributed to research design, gaining ethical approval, recruitment, data collection, data analysis and manuscript writing. Dr. Richardson contributed to data analysis and manuscript writing. Beck Lowe contributed to data analysis and manuscript writing.

## Appendix: Survey Scales and Questions

**Table:**

	Brief stimulus materials	Open-ended question
1	Modified version of the demographic inventory used in other published body image research (Edwards et al., 2016a, 2016b).	Please use this box to explain how different or not your training is at this current time compared to the way it was pre COVID-19.
2	Perceived Choice and Awareness of Self Scale (PCASS). https://selfdeterminationtheory.org/perceived-choice-and-awareness-of-self-scale/	Please use this box to explain how different or not your responses are at this time compared to the way they would have been pre COVID-19.
3	Need Thwarting Scale (NTS) (Costa et al., 2015).	Please use this box to explain how different or not your responses are at this time compared to the way they would have been pre COVID-19.
4	DSM-5 Body Dysmorphic Disorder with Muscle Dysmorphia specifiers (American Psychiatric Association, 2013).	Please use this box to explain further how the current COVID-19 situation has affected your concerns about your appearance.
5	SCOFF (Morgan et al’s., 1999).	Please use this box to explain how the current COVID-19 situation has affected your eating behaviour.
6	Five yes-no response format questions on help-seeking for diagnosed and self-identified body dysmorphic disorder and help-seeking (e.g., Have you ever asked for help from a healthcare professional or support group because you believe you could have Body dysmorphic disorder?).	If you have answered “yes” you have sought help for anything related to your concerns about your appearance, you can use this box to provide us with any additional information about this if you wish.
7	How much has the current COVID-19 situation affected your ability to seek help and support for your concerns about your appearance (visual analogue scale from *not at all* – *totally*)?	Please use this box to explain further how the current COVID-19 situation is affecting your ability to seek help and support for your concerns about your appearance.
8	Six yes-no response format questions on help-seeking for diagnosed and self-identified muscle dysmorphic disorder and help-seeking (e.g., Have you ever asked for help from a healthcare professional or support group because you believe you could have Muscle dysmorphic disorder or muscle dysmorphia?).	If you have answered “yes” you have sought help for anything related to your concerns about your muscle building, you can use this box to provide us with any additional information about this if you wish.
9	How much has the current COVID-19 situation affected your ability to seek help and support for your Muscle Dysmorphic Disorder or muscle building (visual analogue scale from *not at all* – *totally*)?	Please use this box to explain further how the current COVID-19 situation is affecting your ability to seek help and support for your Muscle Dysmorphic Disorder or muscle building.
10	Not applicable	Finally, is there anything else about your concerns about your appearance, body muscularity or any other issues raised in the survey which you think it would be useful for us to know?
